# Survey sampling design in wave 1 of the Global Flourishing Study

**DOI:** 10.1007/s10654-024-01167-9

**Published:** 2025-03-27

**Authors:** R. Noah Padgett, Richard G. Cowden, Manas Chattopadhyay, Ying Han, John Honohan, Zacc Ritter, Rajesh Srinivasan, Byron R. Johnson, Tyler J. VanderWeele

**Affiliations:** 1 Department of Epidemiology, Harvard T.H. Chan School of Public Health, Boston, MA USA; 2 Human Flourishing Program, Harvard University, Cambridge, MA USA; 3 Gallup, Inc., Rockville, MD USA; 4 Gallup, Inc., Ellicott City, MD USA; 5 Gallup, Inc., Ossining, NY USA; 6 Gallup, Inc., San Mateo, CA USA; 7 Gallup, Inc., Princeton, NJ USA; 8 Institute for Studies of Religion, Department of Sociology, Baylor University, Waco, TX USA; 9 School of Public Policy, Pepperdine University, Malibu, CA USA; 10 Department of Biostatistics, Harvard T.H. Chan School of Public Health, MA Boston, USA

**Keywords:** Global, Cross-cultural, Flourishing, Longitudinal, Methodology, Survey sampling

## Abstract

The Global Flourishing Study (GFS) is an international collaboration to develop a publicly accessible data resource to promote global research on human flourishing. These data include over 200,000 participants from 22 geographically and culturally diverse countries and one territory designed to be nationally representative of the adult population. The GFS is intended as a longitudinal panel study with recruitment and empanelment for Wave 1 occurring between April 2022 and December 2023. Future waves of data collection will invite participants to complete a survey annually. The annual survey covers a robust set of measures on well-being, health, social, economic, political, religious, spiritual, psychological and demographic variables. The current paper describes the sampling methodology and weighting approaches used to project the samples to be nationally representative. Details are provided on interviewer training and data collection, probability and non-probability samples, creating weights, design effects, and future data collection stages.

## Introduction

Human flourishing might be considered living in “a state in which all aspects of a person’s life are good, including the contexts in which that person lives” [1, 2]. While there is a sizeable (and growing) body of empirical scholarship focused on understanding and promoting different aspects of human flourishing, a key and unresolved criticism is that such work to date has been Western centric [3]. Henrich et al. [4] argued that most social scientific work has focused on people from nations that may be described as “WEIRD” (i.e., Western, educated, industrialized, rich, and democratic). Although such descriptions are crude as a binary classification of cultures due to the breadth of diversity within nations [5] the criticism is nevertheless important. The cultural bias toward WEIRD contexts has several consequences, including concerns about whether the existing body of empirical literature on human well-being can be used to derive universal principles that are generalizable to many cultural contexts [6]. It is of course not possible to derive unambiguous inferences from WEIRD participants because a complex network of localized ecological forces (e.g., demography, culture, social-structural conditions) influences various dimensions of human life [7].

**Fig. 1:**
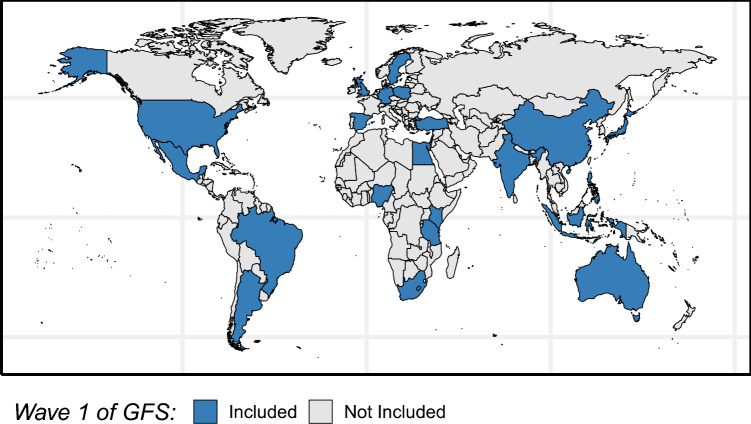
Geographic diversity of countries included in Wave 1 of the Global Flourishing Study. *Note*: Map generate using the ggplot2 package [12] in R [13]

In response to concerns about WEIRD biases in social scientific research, an era of “global scholarship” on human well-being has emerged [3]. The Global Flourishing Study (GFS), a pioneering longitudinal research study, is arguably at the forefront of this era [8]. Guided by a broad and expansive notion of human flourishing [1, 2], the GFS seeks to examine the distribution and determinants of human flourishing in various parts of the world. The study is the largest of its kind, with more 200,000 participants across a culturally and geographically diverse set of countries included in Wave 1 (see Fig. 1). The current paper contributes to the dissemination and open-science commitment of the GFS by providing an overview of the survey development process for the study, a summary of the sampling design, population coverage, weighting approach, response rates, and design effects for Wave 1, and plans for future waves of data collection. The description of the sampling methods and study design are adapted from the methodology report developed by Gallup Inc. [9] and the survey development process reported by Gallup Inc. [10] and Lomas et al. [11].

## Global Flourishing Study overview

### Survey development

Development of the GFS survey occurred over eight distinct phases: (1) selection of core well-being and demographic questions; (2) solicitation of social, political, psychological and demographic questions from domain experts worldwide; (3) Revision of the initial survey draft based on feedback from scholars around the world representing various academic disciplines; (4) modification of question items following input from experts in multinational, multiregional and multicultural survey research; (5) survey draft refinement based on compiled input from an open invitation to comment, posted publicly and sent to numerous listservs; (6) questionnaire optimization with support from Gallup survey design specialists; (7) adaptation of items from an interviewer-administered to a self-administered survey instrument using best practices for web survey design to minimize item non-response, illogical responses and incomplete responses; and (8) confirmation by scholars in several participating countries that translations accurately captured the intended meaning of each question. Further details about the survey development process of the GFS survey can be found in [10, 11].

#### Content coverage of the survey

The aspects of flourishing constitute a broad range of constructs that are related to a person’s well-being. The overall GFS survey includes distinct 109 items in total (43 items in the intake survey and an annual survey of 71 items, with five items shared by both) [11]. A core measure embedded in the survey is the Secure Flourish Index [1], which consists of 12 items that are evenly distributed across six key domains of personal flourishing (i.e., happiness & life satisfaction; physical & mental health; meaning & purpose; character & virtue; close social relationships; financial & material stability); a wide range of measures assessing domains or aspects (e.g., community, political religion, spirituality, socioeconomic factors) that intersect with human flourishing are also included in the survey. The interested reader is referred to Lomas et al. [11] for more information about the survey development process and the full set of survey items, which can also be located in the publicly available codebook (https://osf.io/cg76b).

#### Translation

Wave 1 of the GFS includes 22 countries, namely Argentina, Australia, Brazil, Egypt, Germany, Hong Kong (S.A.R. of China), India, Indonesia, Israel, Japan, Kenya, Mexico, Nigeria, Philippines, Poland, South Africa, Spain, Sweden, Tanzania, Turkey, the United Kingdom, and the United States. The GFS survey was implemented in 36 major languages spoken across these countries, including Afrikaans, Arabic, Assamese, Bengali, Bicol, Cebuano, Chinese, English, German, Gujarati, Hausa, Hebrew, Hiligaynon, Hindi, Igbo, Iluku, Indonesian Bahasa, Japanese, Kannada, Malayalam, Marathi, Odia, Pidgin, Polish, Portuguese, Punjabi, Sotho, Spanish, Swahili, Swedish, Tamil, Telegu, Turkish, Waray, Xhosa, Yoruba, and Zulu. Further details about the countries in which specific language versions of the GFS survey were used can be found in the methodology report appendix (pp. 29–31) [9].

The survey translation process adhered to a modified TRAPD model [14], which stands for translation, review, adjudication, pretesting, and documentation. The translation process that was followed for the GFS survey included (T) a professional translator who translated the survey into the target language using a shared set of notes and guidance about the meaning of specific words, phrases, and concepts; (R) a different professional translator reviewed the translation. This reviewer identified any issues with the translated material, suggested alternative translations, and provided reasoning in English behind their decision for modifications; (A) the original translator received feedback on the disputed translations and accepted or rejected the suggestions. If they disagreed with the reviewer’s edits, the initial translator provided an explanation in English. A third-party reviewer then adjudicated the translation based on the explanation that best aligned with the research objectives; (P) local partners ran a pilot test of the entire questionnaire with at least 10 respondents per language to ensure the accuracy and quality of the translations; and (D) final translations were documented for researchers [15].

In several countries, data collection occurred using a combination of interviewer- and self-administered approaches, depending on the participant’s access to the internet and willingness to complete online surveys. To ensure translation consistency across modes of data collection, a professional translator adapted the final interviewer‑administered translation to reflect the modifications required for the self‑administered version of the survey.

#### Interviewer training

As described by Ritter et al. [9], local field partners employed over 3,000 interviewers in 19 countries to recruit and conduct the first wave of data collection (participants in Hong Kong [S.A.R. of China], Sweden, and the United States were recruited entirely via a web-based approach). Partners were selected based on experience in nationwide survey research studies. They conducted in-depth training sessions with local field staff prior to the start of data collection. Fieldwork teams were assisted by a standardized training manual to ensure consistency and structure. Interviewer training included the following topics: research ethics; protecting participants’ confidentiality; staying safe while in the field; starting the interview; reading survey questions verbatim; handling questions from participants; closed-end and open-end items; skip patterns; interviewing best practices, including probing; respondent selection; household selection and substitution (for face-to-face surveys); and quality control procedures. Field teams trained using the computer-assisted personal interview (CAPI) system for face‑to-face interviews and the computer-assisted telephone interview (CATI) system for telephone interviews, which were employed during fieldwork. These systems ease interviewer burden and facilitate accurate data capture for items such as participant selection in the household, contact data, and skip patterns.

Given the longitudinal research design, interviewer training also focused on accurately capturing the participant contact information required for future recontact. In addition to repeating contact details back to participants, interviewers were trained on double-entry of open-end contact information, which had to match before advancing to the next survey item. Local field partners also emphasized the importance of striking the right balance between friendly persistence in collecting as many forms of contact information as possible and potentially upsetting reluctant participants. Finally, interviewers learned that respondent selection on the annual survey required immediate confirmation of the participant’s name to ensure those who completed the intake survey also completed the annual survey.

### Sampling design and data collection

**Fig. 2:**
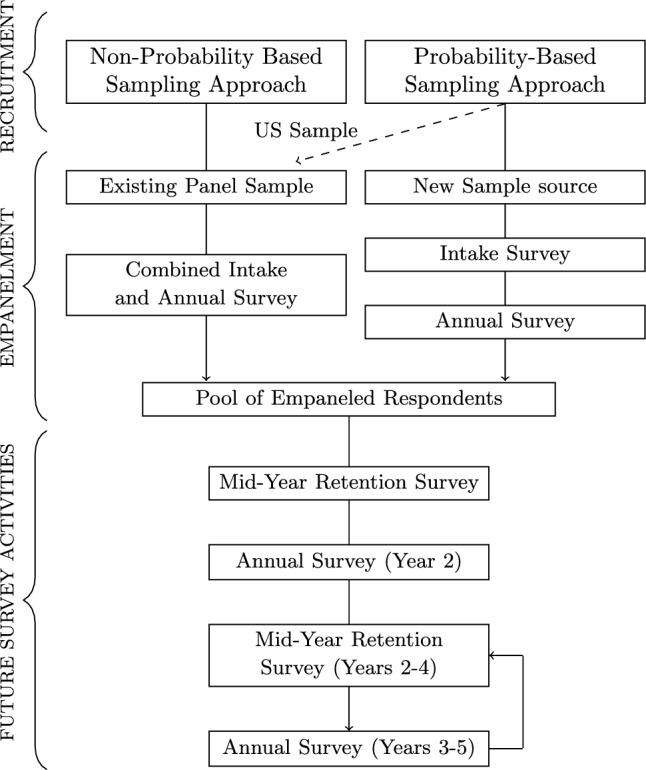
Recruitment and Empanelment in the Global Flourishing Study. *Note*: The United States (US) sample is a subset of the existing Gallup Panel™

The GFS employed various survey methodologies to recruit participants [9]. In most countries, local field partners were guided in implementing a probability-based face‑to-face or telephone methodology to recruit panel members. Recruitment involved an intake survey that mainly gathered basic demographics and information for recontact. Shortly following recruitment, participants received invitations to participate in the annual survey via phone or online. The questions on this latter survey will appear in future yearly waves of data collection to obtain repeated measures. A high-level summary of the recruitment and data collection phases is shown in Fig. 2.

The geographic coverage in each country included in the GFS was the entire country, including rural areas. It represents the entire civilian, non-institutionalized population aged 18 and older. Exceptions included areas where the safety of interviewing staff was threatened, as well as scarcely populated islands and areas that interviewers could only reach by foot, animal, or small boat (although some interviewers reported taking small boats to get adequate population coverage in accordance with the sampling design). Eligibility for participation in the study required the selected participants to have access to a phone or the internet, a practical necessity to help retention. Loss of coverage due to these requirements was < 2% in each country where participants were recruited in person.

Three major sampling frames were used for recruitment in the GFS, namely a probability-based sample, a non-probability-based sample, or a combination of the two [9]. Table 1 summarizes the sampling design within each country. A probability-based sampling approach was used in Egypt, India, Indonesia, Israel, Kenya, Nigeria, Philippines, South Africa, Tanzania, Turkey, and the United States. A non-probability-based sample was recruited in some countries to supplement probability samples so that adequate coverage of population subgroups (i.e., sex, age, region) was achieved. Recruitment and empanelment for Wave 1 of the study occurred between April 2022 and December 2023 [9].

**Table 1:** Global flourishing study sampling summary across countries for Wave 1

Country	Sampling design	Dates of collection	# Participants	Total sample size
Argentina	Probability	11/29/22–11/30/23	3879	6724
	Non-probability	04/11/23–11/14/23	2845	
Australia	Probability	03/21/22–09/26/23	3377	3844
	Non-probability	04/12/23–09/29/23	467	
Brazil	Probability	11/23/22–11/26/23	3793	13,204
	Non-probability	04/11/23–11/07/23	9411	
Egypt	Probability	03/09/23–09/17/23	4729	4729
Germany	Probability	08/11/22–11/16/23	4480	9506
	Non-probability	06/12/23–08/27/23	5026	
Hong Kong (S.A.R. of China)	Non-probability	10/10/23–11/24/23	3012	3012
India	Probability	04/25/23–12/08/23	12,765	12,765
Indonesia	Probability	11/07/22–10/27/23	6992	6992
Israel	Probability	11/07/22–11/23/23	3669	3669
Japan	Non-probability	12/13/22–06/30/23	20,543	20,543
Kenya	Probability	04/13/23–11/21/23	11,389	11,389
Mexico	Probability	10/29/22–12/13/23	2672	5776
	Non-probability	05/09/23–11/20/23	3104	
Nigeria	Probability	05/16/23–11/17/23	6827	6827
Philippines	Probability	04/04/23–01/05/24	5292	5292
Poland	Probability	12/14/22–10/13/23	8444	10,389
	Non-probability	06/10/23–10/16/23	1945	
South Africa	Probability	02/26/23–12/08/23	2651	2651
Spain	Probability	08/17/22–11/08/23	2309	6290
	Non-probability	06/22/23–08/30/23	3981	
Sweden	Non-probability	01/16/23–02/22/23	15,068	15,068
Tanzania	Probability	02/17/23–11/30/23	9075	9075
Turkey	Probability	04/15/23–01/05/24	1473	1473
United Kingdom	Probability	04/26/22–11/20/23	2341	5368
	Non-probability	06/12/23–08/30/23	3027	
United States	Probability	08/04/22–04/04/23	38,312	38,312

Probability-based samples included a mix of face-to-face, telephone, and online data collection modes

Data from Hong Kong (S.A.R. of China) is available in the first wave of data collection. Data from mainland China were not included in the first data release due to fieldwork delays. The first wave of fieldwork in mainland China began in February 2024, and a second wave is expected to occur in November–December 2024. All wave 1 and 2 data from mainland China will be part of the second dataset release in March 2025.

Table 2 summarizes the number of participants who were recruited by different modes in each country. While some countries relied entirely on web-based recruitment (e.g., United States), other countries exclusively used face-to-face recruitment (e.g., Israel, Kenya) or a mix of recruitment methods (e.g., Argentina, Mexico). An additional unique subsample within Nigeria was carried out representative of those without access to a landline, mobile device, or internet. An attempt was made at collecting a similar subsample in India, but implementation was ultimately not completed due to the extremely small proportion that met these requirements there. The weighting approach that was used to ensure that population representative inferences could be obtained in Nigeria is described in more detail within the *Weighting and Design Effects* section below.

**Table 2:** Sample size by mode of data collection employed in the Global Flourishing Study

Country	Recruitment (Intake) survey			Wave 1 annual survey		
	Face-to-face^a^	Telephone^b^	Web^c^	Face-to-face^a^	Telephone^b^	Web^c^
Argentina	1152	2788	2784	0	1451	5273
Australia	0	3844	0	0	0	3844
Brazil	859	2948	9397	0	1265	11,939
Egypt	4729	0	0	0	4508	221
Germany	0	4504	5002	0	0	9506
Hong Kong (S.A.R. of China)	0	0	3012	0	0	3012
India	12,765	0	0	0	12,549	216
Indonesia	6139	853	0	0	3620	3372
Israel	3669	0	0	0	2743	926
Japan	0	542	20,001	0	542	20,001
Kenya	11,389	0	0	0	9917	1472
Mexico	1250	1535	2991	0	1559	4217
Nigeria	6827	0	0	550^d^	5609	668
Philippines	4620	672	0	0	4174	1118
Poland	8444	1945	0	0	625	9764
South Africa	2651	0	0	0	2010	641
Spain	0	2339	3951	0	0	6290
Sweden	0	0	15,068	0	0	15,068
Tanzania	9075	0	0	0	8790	285
Turkey	333	1140	0	0	840	633
United Kingdom	0	2358	3010	0	0	5368
United States	0	0	38,312	0	0	38,312

^a^Face-to-face interviews used the computer-assisted personal interview (CAPI) system; ^b^Telephone interviews used the computer-assisted telephone interview (CATI) system; ^c^Web surveys used the computer-assisted web interview (CAWI) system; and ^d^Nigerian face-to-face annual subsample is weighted to be representative of the population of Nigeria without access to a landline, mobile device, or internet

#### Probability-based samples

For face-to-face interviews, the selection of probability-based samples was carried out by selecting sampling units stratified by population size, urbanicity and/or geography, and clustering [9]. This complex design varied by country, leading to a different number of sampling stages depending on the country. A stratified single-state or multi-stage cluster design was employed in countries where detailed population information was available from a recent census or other reliable source. Sampling units were selected using probabilities proportional to population size for each sampling stage down to the cluster, with a fixed number of interviews completed within each cluster. Countries with more limited population information (e.g., population data only at the province or district level) utilized a stratified multi-stage cluster design. Primary sampling units (PSUs) were selected during the first sampling stage using probabilities proportional to size, and units at subsequent stages were selected using simple random sampling; no more than four clusters per PSU were used in the last stage of sampling. When a multi-stage sampling design was employed, the pre-determined clusters defined the geographic region from which households and participants were ultimately selected, where interviewers, starting from an address or structure closest the random coordinate starting point that was pre-selected by a field manager, were trained to select every third household to invoke a pseudo-random route. The interviewer randomly selected a participant within each household using the computer assisted personal interview (CAPI) system. If the randomly selected household member was unavailable for the duration of the data collection period, or unwilling to participate in the study, a different household was selected. However, if the randomly selected household member was unavailable due to being at work or out shopping, then more contact attempts were made at varying days of the week and times of day (up to three contact attempts).

For telephone interviews, the selection of participants was carried out using random-digit dialing or a nationally representative list of phone numbers [9]. When applicable, either a dual sampling frame of landline and mobile phones was used or a mobile phone only frame was used. Landline samples were stratified by region, whereas mobile phone samples were primarily stratified by mobile service providers. Brazil was the only exception in that the mobile sampling frame was stratified by region. The size of regional samples was determined to be proportional to the size of the adult population aged 18 or older, and a random participant within each household was obtained using one of the standard methods—enumeration of adults 18 or older and selecting one at random for face-to-face collection or asking for the person who had the next birthday among eligible adults 18 or older for interviews completed over telephone. Interviewers attempted contact over several different days and times, with at least five attempts at contact, to reach the intended participants to complete the interview. When successful contact was made, the telephone intake interview was aided by a computer assisted telephone interview (CATI) system, which was also used to help guide interviewers through the annual survey to maintain valid skip logic when necessary.

Data collection in the United States used the Gallup Panel™, a probability-based, nationally representative panel for which all members are recruited via address-based sampling or random-digit-dial methodology [9]. All online members of the Gallup Panel™ received up to five invitations to complete a single survey that included all intake and annual items together.

For participants from a country other than the United States who completed the intake survey as part of a probability-based sample, retention efforts to ensure completion of the annual survey involved a multipronged approach. As described above, web surveys used multiple outreach attempts across various recontact channels increased the chances that a participant received an invitation and reminders to participate in the annual survey. Receipt of the invitation is a necessary but insufficient condition for ensuring participation. Participants assigned to the annual survey received a welcome message shortly after completing the intake survey that provided more context about the study and a link to a website, which included additional information on frequently asked questions and a way to contact recruiters. For participants assigned to complete the annual survey over the telephone, interviewers received training on refusal conversion tactics. Additionally, the introduction script included a section of short responses to frequently asked questions and concerns. Interviewers applied similar cooperation tactics when attempting to recontact surveys with respondents assigned to the web survey who had not participated after the initial five-invite design. As a small token of appreciation for their time, eligible participants who completed the annual survey received a gift card or mobile top‑up worth roughly $5.

#### Non-probability based samples

The samples from Hong Kong (S.A.R. of China), Japan, and Sweden relied exclusively on existing web panels for recruitment, leading to a non-probability-based sample [9]. Data collection in Hong Kong (S.A.R. of China) and Sweden recruited a non-probability sample obtained through online opt-in panels. A small proportion of the sample in Japan ($N \approx 500$) was drawn from a probability frame, but the sample was insufficient to project that sample to the target population.

Data collected in Hong Kong (S.A.R. of China), Japan, and Sweden were based solely on a non-probability sample, so creating true sampling weights is not possible; however, as discussed in greater detail in the *Weighting and Design Effects* section, for Sweden, pseudo-sampling weights based on propensity scores methods were constructed while for the other two countries a baseweight of 1 was assumed and a more detailed cross-classified population targets were used so that weighted estimates are approximately representative of the population.

#### Combined probability and non-probability samples

Data collection in Argentina, Australia, Brazil, Germany, Mexico, Poland, Spain, and the United Kingdom consisted of a combination of two samples: (1) a probability-based sample selected with a face-to-face sampling methodology, or a phone sampling methodology or a combination of the two (both of which are described in more detail in the *Probability-based samples* subsection); and (2) a non-probability-based sample collected through web panels.

Data collection in Germany and the United Kingdom used high-quality, commercially available, or proprietary third-party panels put together using opt-in methods [9]. Data collection in Argentina, Brazil, Mexico, and Spain recruited directly from a proprietary affiliate network that is the foundation of many commercial panels. This network comprises media entities that offer access to internet and mobile users across the gamut of digital marketing channels, including, but not limited to, social media marketing, email, video, mobile, blogs, and display.

Quotas for age, gender, region, and education were set and monitored during fieldwork in countries that use existing web panels to ensure adequate representation of the population. Participants who were recruited from these panels completed a single, combined survey. Population coverage in Germany, United Kingdom, and Spain excluded the offline population for these countries. Probabilities samples in Latin American countries (Argentina, Brazil, Mexico) reached the offline population with the inclusion of respondents completing the annual survey via telephone surveys assisted by CATI.

#### Ethics and respondent confidentiality

Ethical approval was granted by the institutional review boards at Baylor University (IRB Reference #: 1841317) and Gallup (IRB Reference #: 2021-11-02), and all participants provided informed consent. Individuals may withdraw from participating in the study at any point. Consistent with European Union regulations, respondents from a country in this region (e.g., Germany, Spain, Sweden) can additionally request that their data be completely removed from subsequent waves of the GFS. Respondent confidentiality is maintained by giving a pseudo-random identification number that is kept separate from the data that researchers can access. A public use dataset has been created and will be publicly available in February 2025, and can be accessed by anyone submitting a pre-registration to the Center for Open Science prior to then, but this dataset excludes individual identifying information such as location (latitude and longitude are rounded to the nearest degree) and language of assessment. Individual researchers with appropriate institutional review board approval may be able to access a restricted use dataset that contain these more sensitive types of data.

### Post-collection data quality evaluation

Each country dataset underwent a rigorous quality assurance process by survey type (intake or annual) and mode (telephone, face-to-face, or web). Gallup’s regional directors of survey research verified that the sampling plan was followed, confirmed the data were nationally representative, and reviewed the data for consistency, reliability, and validity by interviewer and region [9]. They also checked response consistency across demographic items on the intake and annual survey. After the regional directors reviewed the data, quality control analysts at Gallup performed additional validity reviews. The data were centrally aggregated and cleaned, ensuring that correct variable codes and labels were applied. The data were then reviewed by a team at Gallup in detail for completeness, accuracy, and logical consistency.

#### Face-to-face and telephone survey administration

To ensure interviewers followed the methodology and administered the survey properly, vendors were required to conduct in-field validations for a percentage of face‑to‑face and telephone interviews. Face-to-face interviews were validated by supervisor accompaniment, in-person recontact, phone recontact, or listening to recorded interviews. Telephone interviews were validated by live listen-ins or audio recordings.

At least 30% of completed face-to-face interviews were validated using accompanied interviews, in-person recontacts, or telephone recontacts. Validations were distributed across all regions and interviewers. The supervisor/validator evaluated the interviewer’s performance in implementing the survey methodology, including starting point selection, random route procedure, correct disposition code usage, participant selection, and proper survey administration (e.g., reading each question verbatim, not leading the respondent, etc.). Globally, approximately 25% of recruitment telephone interviews and 30% of baseline telephone interviews were validated. These validations confirmed that the interview was completed, methodological standards were followed (e.g., participant selection), and the survey was administered appropriately [9].

Additionally, interviewer productivity metrics and metadata were tracked throughout data collection [9]. These data will be made available as part of the sensitive data release starting in August 2024, and researchers will need to obtain IRB approval prior to accessing these data. The CAPI and CATI platforms used in the project provided monitoring tools for regularly evaluating collected data. Quality control analysts and in-country partners used these tools to ensure that completed interviews were valid and adhered to the methodology. Holistic reviews at regular fieldwork intervals covered a range of quality control procedures and aimed to identify suspicious patterns in the data (e.g., anomalies in interview duration, location, household selection, and participant selection). Flagged interviews were further investigated and validated, and surveys with quality issues were removed and replaced (when possible). At the end of fieldwork, final data vetting by Gallup ensured the validity, reliability, and accuracy of the collected data. The overall retention rates for each country after non-response and quality checks are provided Table 3.

**Table 3:** Availability of variables for non-response and post-stratification adjustments for each country in wave 1 of the Global Flourishing Study annual survey

Country	Variables used	Target source
Argentina	Age × Gender × Region, Education, Employment	Population and Housing Census 2010, National Institute of Statistics and Censuses (INDEC)
Australia	Age × Gender × Region, Age × Gender × Education, Age × Gender × Employment	2021 Census, Australian Bureau of Statistics
Brazil	Age, Education, Region × Gender	2010 Census, Brasilian Institute of Geography and Statistics (IBGE)
Egypt	Age, Education, Age × Gender × Region	Census 2017
Germany	Age × Gender × Region, Education, Employment, Age × Gender × Marital	Eurostat 2021; Federal Statistical Office 2019
Hong Kong	Age × Gender × Region, Age × Gender × Education	2022 data from Census and Statistics Department of Hong Kong
India	Age × Gender × Region, Age × Gender × Education, Age × Gender × Religion, Age × Gender × Urbanicity	India Census 2011, Office of the Registrar General & Census Commissioner, India
Indonesia	Age × Gender × Region, Education	Indonesia Population Census 2020 and Census 2010, National Bureau of Statistics
Israel	Age × Gender × Region, Age × Gender × Marital, Age × Gender × Education × Race, Gender × Ethnicity × Employment	Statistical Yearbook 2023
Japan	Education, Region, Age × Sex × Region × Employment, Age × Sex × Region × Marital Status	2020 Population Census
Kenya	Age × Gender × Region, Education	Kenya Population and Housing Census 2019, Kenya National Bureau of Statistics
Mexico	Age × Gender × Employment, Gender × Education × Region	Population Census 2020, Mexico’s National Bureau of Statistics (INEGI)
Nigeria	Age, Gender, Education, Region, Main Supplement (have any access to landline, mobile, internet vs not)	DHS 2018
Philippines	Age × Gender × Education, Age × Gender × Region, Age	Philippines Population and Housing Census 2020, Philippines Statistics Authority
Poland	Age × Gender × Region, Age × Gender × Employment, Education	Eurostat 2021 and Central Statistical Office of Poland, 2019
South Africa	Age × Gender, Age × Region, Education	U.S. Census, IDB 2022 and DHS 2016
Spain	Age × Gender × Region, Age × Gender × Education, Age × Gender × Employment, Age × Gender × Marital	Eurostat 2021 and Population and Housing Census 2021
Sweden	Age × Gender, Education, Region	2018 population estimates, Statistics Sweden
Tanzania	Age × Gender × Region, Age × Gender × Education	Census 2022 and DHS 2022
Turkey	Age × Gender, Education, Region, Employment, Marital Status	Address Based Population Registration System, 2022; National Education Statistics Database 2008–2022; Household Labour Force Survey as of January 2021; Population and Housing Census 2021, TurkStat
United Kingdom	Age × Gender × Region, Education, Age × Gender × Employment	England and Wales Census 2021, Northern Ireland Census 2021, National Records of Scotland—Mid Year Population Estimates 2021
United States	Black: Age × Gender × Education, Region	American Community Survey 2021 5-year estimates
	Non-Black: Age × Gender × Education, Region	
	Hispanic: Age × Gender × Education, Region	

Under Variables Used, an “×” represents the use of cross-tabulation of the characteristic

#### Online survey

Quality control was informed by the recent American Association for Public Opinion Research task-force report [16]. In addition to the standard quality control processes used to evaluate face‑to‑face and telephone data, the following quality control procedures were applied to completed online surveys. First, response metadata (i.e., response digital fingerprint) was evaluated to ensure surveys weren’t completed by bots, responses came from valid devices, and that all responses were unique. Duplicates or completed online surveys that were not from valid digital sources were removed in line with recommendations for quality assurance [17]. Next, responses were evaluated for inconsistency in responses to closely related items. Such cases were removed when other quality issues were present, but a seemingly inconsistent response by itself was not sufficient for removal. Next, participants who consistently selected a single response category for items within a question block were flagged as potentially problematic (i.e., straight-line responses). Such response patterns are plausible for valid cases, so such cases were only removed if other quality issues were present. Lastly, the total time required to complete the survey was checked against a minimum threshold of time required to read through the questions included in the survey [18]. A common threshold of 4 min was used across all countries for the annual survey, and a modified thresholds of 5 min was used for participants taking the combined intake and annual survey. Taken together, these response characteristics were used to evaluate each online survey response to ensure that the final cases included in the annual survey represent valid responses by participants.

#### Handling seemingly inconsistent responses

Although the above data quality checks were used to ensure the final dataset available for use by researchers has a minimal number of inconsistent and invalid responses, the dataset may nevertheless may contain seemingly inconsistent responses. A seemingly inconsistent response could be due to various reasons, such as misinterpretation of question by the respondent, temporary loss of attention by respondent, accidental data entry error by the interviewer, or another reason that is unknown to us but is internally logically consistent for the respondent. Adopting a holistic approach to quality control at the case level, a case is not thrown out of the dataset due to one or two perceived response inconsistencies. An ostensibly inconsistent case would require additional burden of proof before being discarded; otherwise such discarding has the potential to create unintended consequences related to disproportionate removal of certain types of respondents. Researchers may, however, still come across responses that seem logically inconsistent but may still be valid responses by the respondent.

To illustrate this, consider responses to the items *Relationship with Mother* (wording: Please think about your relationship with your mother when you were growing up. In general, would you say that relationship was very good, somewhat good, somewhat bad, or very bad? If you didn’t know your mother or if she died, select “Does not apply.”) and *Love from Mother* (wording: In general, did you feel loved by your mother when you were growing up? If you didn’t know your mother or if she died, select “Does not apply.”), it would be seemingly inconsistent to select “Does not apply” to only one of these items. However, the circumstances that each respondent was considering while responding to these items may vary. For example, a respondent may endorse “Does not apply” to the *Love from Mother* item because they equated “love” with “romantic love” or some form of love they felt wasn’t applicable to a familial relationship, whereas they perhaps did in fact have a relationship with their mother. Other explanations are also possible. The translation of the concept of “love” was challenging for this item, which is documented and discussed at length in the companion paper on translation and cognitive interviewing [11].

In practice, there are several approaches researchers can take to handling such cases. One possibility is to recode any response of “Does not apply” as a missing value and then handle missing data using a method appropriate to their analysis (e.g., multiple imputation, full-information maximum likelihood, etc.). Another approach could be to use “Does not apply” as a category of variable to evaluate whether those who respond “Does not apply” differ from a suitable reference group for the chosen analysis. The appropriate method for handling seemingly inconsistent responses will depend on context and purpose of the analysis. Caution should be exercised so that unintended measurement errors are not introduced by overcleaning.

### Weighting and design effects

Case weighting was used to ensure samples were nationally representative of each country and was intended to be used for calculations within a country [9]. The methodologies and sources used to build country samples varied significantly. These differences required a customized approach to data weighting that incorporates probability and non-probability samples [19]. Final country weights only included respondents who completed the intake and annual surveys. To produce the final respondent weight, an initial weight was constructed for those who completed the intake survey. Further calibration on the initial weight occurred when an additional layer of post-stratification was adjusted for non-response on the annual survey. All non-response/post-stratification adjustments matched variables based on the marginal distribution of each matching characteristic. Countries where more detailed cross-classified population targets were used are shown in Table 3. The methods used for weighting intake surveys can be grouped into three main approaches based on the type of sample sources.

#### Approach 1: Probability samples only

Countries using probability-based samples only: Egypt, India, Indonesia, Israel, Kenya, Nigeria, Philippines, South Africa, Tanzania, Turkey, and United States. In countries where data were collected using a single probability-based sample (e.g., Israel), sampling weights were constructed as the inverse of the selection probability for inclusion in the sample based on each country’s sampling methodology [9]. For face-to-face sampling methodology, overall selection probability accounted for selection probabilities at different stages of selection, including disproportionalities in allocation, selection of primary sampling units, selection of secondary sampling units (if applicable), household selection within the ultimate cluster, and selection of one eligible respondent within the household. The creation of sampling weights for probability only samples depended on whether households were sampled via a random-digit-dialing or pseudo-random route (see *Probability-based samples* subsection).

Under the random-digit-dialing phone sampling methodology, base sampling weights accounted for the selection of telephone numbers from the respective frames and corrected for unequal selection probabilities due to the selection of one adult in landline households and for dual users coming from both the landline and mobile frame [20, 21]. The base sampling weight for case $i$ is

$$ w_{i} = \left[ {\left( {\frac{{S_{LL} }}{{F_{LL} }} \times \frac{1}{{AD_{i} }} \times LL_{i} } \right) + \left( {\frac{{S_{CP} }}{{F_{CP} }} \times CP_{i} } \right) - \left( {\frac{{S_{LL} }}{{F_{LL} }} \times \frac{1}{{AD_{i} }} \times LL_{i} \times \frac{{S_{CP} }}{{F_{CP} }} \times CP_{i} } \right)} \right]^{ - 1} , $$

where $S_{LL} =$ total count of landline completes in corresponding landline; $F_{LL} =$ the total count of phone numbers in the landline frame in the corresponding stratum; $S_{CP} =$ the total count of mobile completes in each mobile stratum; $F_{CP} =$ the total count of mobile phone numbers in the corresponding stratum in the cell frame; $AD_{i} =$ number of adults in household $i$; $LL_{i} = 1$ if respondent has a landline phone, otherwise $LL_{i} = 0$; and $CP_{i} = 1$ if respondent has a cell phone, otherwise $CP_{i} = 0$ [9].

Under the face-to-face sample methodology, the base sampling weights similarly accounted for unequal probability of inclusion, but with a slightly difference in sampling frame. The general method used across countries to construct the base sampling weights assuming a single stage design is

$$ w_{i} = \left[ {\left( {\frac{1}{{HR_{ikh} }}} \right) \times \left( {\frac{{HH_{kh} }}{{\mathop \sum \nolimits_{\forall k} HH_{kh} }}} \right) \times \left( {\frac{{P_{h} n_{kh} }}{{\mathop \sum \nolimits_{\forall h} n_{kh} }}} \right)} \right]^{ - 1} , $$

where $HR_{ikh} =$ total number of adults in household $i$ in PSU $K$ in stratum $H$; $HH_{kh} =$ number of households interviewed in PSU $K$ in stratum $H$; $\sum\nolimits_{\forall k} H H_{kh} =$ total number of households in PSU $K$ in stratum $H$; $P_{h} =$ number of PSUs sampled in stratum $H$; $n_{kh} =$ population size of PSU $K$ in stratum $H$; and $\sum\nolimits_{\forall h} {n_{kh} } =$ total population size of stratum $H$ [9]. The formula is adjusted accordingly for different sampling designs.

Once the abovementioned base sampling weights were constructed, weights were post-stratified to adjust for non-response and match existing known target population totals (e.g., auxiliary data from a country-level census). Variables used for non-response/post-stratification adjustments included age, gender, education, region, employment status, marital status, and other variables that possibly vary by country depending on the availability of a reliable secondary source. Table 3 lists out, for each country, which variables were used.

The resulting distribution of post-stratified weights was highly skewed with some extreme weights; these extreme weights were trimmed to reduce estimator variance [22]. Trimming was conducted at both ends of the distribution of weights. The resulting minimum/maximum weight (i.e., trim points) generally fell between the 1–5%- and 95–99%-tiles. The weights were then redistributed across the remaining sample. The trim points were determined based on a tradeoff between bias (generally < 1% difference for age, gender, region, urbanicity and less than 3% for education) and variance as measured by design effect (taking into consideration just the variability in weights). For the purpose of weight calibration, the design effect measured by variability in weights where preferred design effect were below 2.

In several countries, data were collected using more than one probability-based sample (e.g., Indonesia), where the weighting was initially conducted separately for each sample. A combined case weight was then obtained using a composite weighting procedure to project the final combined weight to the target population of adults [23]. The design effects associated with different sample sources are described below.

*Design effects and combined weights.* For illustrative purposes, consider the multi-sample source data collected in Indonesia that consisted of face-to-face and telephone recruitment. Let $w_{i}$ represent the weight assigned to case $i = 1,2, \ldots , n_{1}$ from the face-to-face sample and let $w_{j}$ represent the weight assigned to case $j = 1,2, \ldots ,n_{2}$ from the telephone sample. The number of complete face-to-face and telephone samples are $n_{1}$ and $n_{2}$, respectively. The corresponding effective sample sizes under each recruitment methods are

$$ n_{1}^{*} = \frac{{n_{1} }}{{deff_{1} }}, $$

$$ n_{2}^{*} = \frac{{n_{2} }}{{deff_{2} }}. $$

where $deff_{1}$ and $deff_{2}$ are the design effects associated with face-to-face and telephone sampling, respectively [9]. The design effects were estimated using Kish’s method [24], where the design effects are approximated by

$$ deff_{1} = n_{1} \times \frac{{\mathop \sum \nolimits_{\forall i} \left( {w_{i}^{2} } \right)}}{{\left( {\mathop \sum \nolimits_{\forall i} w_{i} } \right)^{2} }},\quad {\text{for}}\;\;i = 1,2, \ldots ,n_{1} $$

$$ deff_{2} = n_{2} \times \frac{{\mathop \sum \nolimits_{\forall j} \left( {w_{j}^{2} } \right)}}{{\left( {\mathop \sum \nolimits_{\forall j} w_{j} } \right)^{2} }},\quad {\text{for}}\;\;j = 1,2, \ldots ,n_{2} $$

The design effects associated with each country are reported in Table 4. A total design effect was estimated for each country and a design effect specific to each mode of data collection for the annual survey (web survey, telephone interval, and face-to-face for Nigerian subsample). In all but one country (the United States) the total design effect was at most 2.0, and in the United States, the design effect was 5.49.

**Table 4:** Global Flourishing Study annual survey design effects—variability in weights—associated with each country

Country	Web	Telephone	Total
Argentina	2.01	1.87	1.99
Australia	1.58	–	1.58
Brazil	1.81	1.50	1.79
Egypt	1.46	1.44	1.44
Germany	1.65	–	1.65
Hong Kong	1.93	–	1.93
India	1.55	1.43	1.43
Indonesia	1.52	1.61	1.59
Israel	1.42	1.31	1.34
Japan	1.38	1.29	1.38
Kenya	1.42	1.49	1.48
Mexico	1.77	1.47	1.68
Nigeria*	1.59	1.93	1.97
Philippines	1.53	1.57	1.56
Poland	1.91	1.83	1.91
South Africa	1.52	1.77	1.71
Spain	1.79	–	1.79
Sweden	1.48	–	1.48
Tanzania	1.45	1.50	1.50
Turkey	1.36	1.45	1.49
United Kingdom	1.97	–	1.97
United States	5.49	–	5.49

^*^Nigerian face-to-face subsample has a design effect = 2.01. Web and telephone surveying for annual survey, and Total represents the design effect (variability in weights) for the entire country sample combined. All design effects were estimated using Kish’s method based on the final GFS sample that is openly available. All reported design effects are based on the published non-sensitive GFS wave 1 dataset—estimated design effects reported in the Gallup methodology report [9] can differ because those estimates include participants who only responded to the intake survey

The final weights are computed adjusting for the effective sample size of each sample source, resulting in weights

$$ w_{i}^{*} = \left( {\frac{{n_{1}^{*} }}{{n_{1}^{*} + n_{2}^{*} }}} \right) \times w_{i} , $$

$$ w_{j}^{*} = \left( {\frac{{n_{2}^{*} }}{{n_{1}^{*} + n_{2}^{*} }}} \right) \times w_{j} . $$

*A note on the Nigerian sample.* In Nigeria alone, the intake sample was split into two parts—one to cover households with individuals having access to a landline, mobile phone, or the internet, and a small number of PSUs to represent individuals living in households that do not have access to a landline, mobile phone, or the internet. Each sample was weighted separately and projected to the corresponding target population. Targets for the population without access to landline or a mobile phone were derived from the latest Demographic and Health Survey in Nigeria [25]. The resulting weighted samples were combined where 90% of sample came from sources with access to telephones or internet and 10% of the sample came from sources not having access to telephone nor internet. This latter sample constitutes what we call a “hard-to-reach” population that is less often included in large scale surveys and studies.

#### Approach 2: Non-probability samples only

Countries and territories using a non-probability sample only: Hong Kong (S.A.R. of China), Japan, and Sweden. Data collection in Hong Kong (S.A.R. of China) and Sweden only used a non-probability sample obtained through online opt-in panels [9]. A small proportion of the sample in Japan was drawn from a probability frame, but it was insufficient to project that sample to the target population. For the purposes of constructing weights, all records in Japan were treated as non-probability samples. Since the selection probability of inclusion in the final sample is unknown, pseudo-base sampling weights were constructed using propensity weighting [19, 26]. The Sweden sample relied solely on propensity weighting. Hong Kong and Japan samples utilized a baseweight of 1 followed up with raking with detailed population targets (see Table 3). This approach estimated the probability of inclusion in the panel frame and generated respondent-level survey weights for subsequent analysis, see pg. 16–17 of [16].

A limitation of solely using a non-probability sample is the need to have an auxiliary probability-based sample from the same country with a subset of identical items [16]. Table 3 provides details on which sources were used provide the auxiliary information needed. To implement propensity weighting, a combined dataset stacked the reference sample with the non-probability sample. A binary variable assigned records from the reference survey a value of “0” and records from the non-probability sample a value of “1”. This binary indicator served as the dependent variable in a weighted logistic regression model. Auxiliary variables such as age, gender, region, measured in both samples (reference and non-probability) and likelihood of selection into the probability sample served as possible predictors. A model selection technique identified the most suitable model, which was used to generate a propensity score representing the predicted probability that a record came from the non-probability sample. The inverse of this predicted probability was the pseudo-selection probability weight assigned to each unit in the non-probability sample.

These weights were then post-stratified to balance sample demographics to known target population totals obtained from country-level census data [9]. Variables used included age, gender, education, region, employment status, marital status, and any other variable for which reliable data were available. There were some differences in the variables used for post-stratification adjustment by country (see Table 3). After post-stratification weighting, the distribution of the final weights was examined and extreme weights were trimmed, where necessary, to minimize the effect of large weights on the variance of estimates.

*A note on Japan and Hong Kong (S.A.R. of China).* In Japan and Hong Kong (S.A.R. of China), no reference was captured to calculate the pseudo-selection weights described earlier. Therefore, the weighting process started with a base weight of 1, and the sample was post-stratified using detailed cross-classified cells. For Hong Kong, post-stratification was based on age by gender by education and age by gender by region. For Japan, post-stratification was based on the cross-classified cells of (1) region, age, gender, and employment status and (2) region, age, gender, and marital status, in addition to education and prefecture.

#### Approach 3: A combination of probability and non-probability samples only

Countries using both probability and non-probability samples: Argentina, Australia, Brazil, Germany, Mexico, Poland, Spain, and the United Kingdom [9]. Combining probability and non-probability samples increases population coverage [16, 27], and some evidence exists that such hybrid complex sampling designs reduce sampling variability [28, 29].

Each probability-based sample was weighted using the weighting steps (sampling weight calculation and post-stratification) outlined in the *Approach 1: Probability samples only* subsection. The non-probability sample applied the weighting steps (pseudo-sampling weight calculation and post-stratification) outlined in the *Approach 2: Non-probability samples only* subsection, using the weighted probability sample as the reference sample. Then, the two parts were combined using composite weighting procedures to project the final combined sample to the target population of adults aged 18 years or older living in each country (see *Design effects and combined weights* subsection for more details).

### Response rate

The response rate was calculated for intake surveys and annual surveys based on the recommendations of American Association for Public Opinion Research [30] for reporting transparency of the survey methodology quality. Although a high response rate reduces the risk of non-response bias [31], a low response rate does not necessarily lead to non-response bias in results [32–35]. The response rates were calculated for face-to-face, telephone, and web recruitment (methods described below), but the response rate could not be calculated for non-probability-based samples due to the lack of information about the sampling frame. In countries utilizing a combination of recruitment methods, a combined response rate was created by weighting the average response rate according to the proportion of recruits from each sample source.

Calculated response rates for face-to-face recruitment follows

$$ RR = \frac{I}{{I + P + R + NC + O + e\left( {UH + UR + UO} \right)}}, $$

where $I =$ total number of complete interviews (defined as responding to at least 75% of survey items); $P =$ total number of partial interview (define as responding to less than 75% of survey items); $R =$ number of refusals; $NC =$ number of non-contacts; $O =$ number of non-response due to some other reason; and $e\left( {UH + UR + UO} \right)$, which encompasses the estimated proportion of cases with unknown eligibility that are eligible, with $UH =$ unknown if household is occupied, $UR =$ unknown if sampled unit is eligible or if household contains an eligible respondent, and $UO =$ unknown if eligible due to some other reason [9].

Calculated response rates for telephone recruitment follows

$$ RR = \frac{I}{{I + P + R + NC + O + \left( {e_{1} \times e_{2} \times UH} \right) + \left( {e_{1} \times UO} \right)}}, $$

where $e_{1} =$ percent of known-residential cases estimated to have eligible respondents; $e_{1} =$ percent of known-residential cases estimated to have eligible residential; and $UH$ and $UO$ are approximated [9].

The Wave 1 annual survey response rate was calculated by multiplying the weighted average response rate above by the ratio of complete responses to the annual survey over the total number of recruited panel members. The intake and Wave 1 annual survey response rates are provided in Table 5. For Hong Kong (S.A.R. of China), Japan, Sweden, and the United States, web-based intake and Wave 1 annual surveys were combined and administered simultaneously and thus the implied Wave 1 Annual Survey response rate in those countries is 100%.

**Table 5:** Response rates for the Global Flourishing Study by country

Country	Sample-type	Recruitment (Intake) survey		Wave 1 Annual survey	
		# Complete	Response rate	# Complete	Response rate
Argentina	Probability	9398	10.5	3879	41.3
	Non-probability	2905	^a^	2845	97.9
Australia	Probability	5539	2.4	3377	61
	Non-probability	677	^a^	467	69
Brazil	Probability	10,257	13.3	3793	37
	Non-probability	9437	^a^	9411	99.7
Egypt	Probability	7500	55	4729	63.1
Germany	Probability	10,941	2.5	4480	41
	Non-probability	5141	^a^	5026	97.8
Hong Kong (S.A.R. of China)	Non-probability	3012	^a^	3012	100
India	Probability	28,644	68.9	12,765	44.6
Indonesia	Probability	11,808	54.6	6992	59.2
Israel	Probability	5507	55.8	3669	66.6
Japan	Non-probability	20,543	^a^	20,543	100
Kenya	Probability	14,993	63.7	11,389	76.1
Mexico	Probability	9896	20.5	2672	27
	Non-probability	3244	^a^	3104	95.7
Nigeria	Probability	13,849	79.2	6827	49.3
Philippines	Probability	13,995	42.6	5292	37.8
Poland	Probability	11,013	71.1	8444	76.7
	Non-probability	3217	^a^	1945	60.5
South Africa	Probability	11,035	82	2651	24
Spain	Probability	5446	3.6	2309	42.4
	Non-probability	4117	^a^	3981	96.7
Sweden	Non-probability	15,068	^a^	15,068	100
Tanzania	Probability	10,997	70.9	9075	82.5
Turkey	Probability	5414	19.2	1473	27.2
United Kingdom	Probability	5380	1.8	2341	43.5
	Non-probability	3153	^a^	3027	96
United States	Probability	38,312	^a^	38,312	100

^a^Response rate for non-probability-based samples was not possible due to lack of information about sampling frame

### Strengths and limitations of wave 1

Wave 1 of the GFS has several strengths and limitations researchers should be aware of when interpreting results. A notable strength is that these data represent a large and diverse sample that is weighted (using the methods described in this paper) to be approximately nationally representative of the general adult population in each of the 22 countries and territories, leading to broad population coverage, along with broad survey coverage of numerous constructs. Another strength of the GFS is the use of a mid-year retention survey to help maintain contact with respondents to reduce drop out and utilize modified or new survey items that cannot be accommodated by the annual survey.

However, several limitations associated with Wave 1 of the GFS should be noted. The time of year data were collected differed across countries, which could lead to seasonality effects that make direct comparisons between countries more challenging. Although each country was weighted to be nationally representative, the respondent level weights are dependent on the quality of the external data source used as target population counts. These targets are documented in Table 3, along with the stratification characteristics used for each country. Additionally, using a different weighting approach for each country leads to complexity about how to interpret heterogeneity across countries. For example, if the weighted mean response to the item *Happy* (“In general, how happy or unhappy do you usually feel?”; a Likert-type response scale of Extremely Unhappy [0]—Extremely Happy [10]) varies across countries, there are several possible sources of this variation. In addition to substantive differences, sources of heterogeneity in the weighted means might also include seasonality effects, differences in interpretation of items or response scales, differences due to quality of translation, differences in mode of data collection, differences in the process and variables used for constructing respondent level weights, and other possible reasons depending on the specific construct of interest.

There are other limitations of the GFS that will apply beyond Wave 1. For example, the GFS uses a self-report survey that assesses most constructs using a single item, which means that the GFS survey may be somewhat limited in its conceptual coverage of the constructs that are measured. Researchers will need to keep these considerations in mind as they process and interpret results using Wave 1 (and subsequent waves) of the GFS.

### Ongoing and future waves

A short retention survey is being administered between Wave 1 and Wave 2 data collection. The objectives of this retention activity are to remind the participant about their involvement in GFS, update contact information, collect responses for a few additional substantive survey items, and to try to ensure retention. This activity will be undertaken only for probability‑based samples, with the additional substantive items for other samples administered instead in the Wave 2 survey. Wave 2 data collection will occur roughly one year following Wave 1. The survey will include the same set of items fielded in Wave 1, producing longitudinal data that can be used to track within-subject changes over time. Additionally, subsequent waves of the GFS may include additional recruitment of new cohorts as funding permits.

## Concluding remarks

The GFS represents a direct response to growing calls that have been made to reshape the Western-centricity of social scientific evidence on human well-being through rigorous research that is more inclusive and representative of the global population [4, 6]. Although the GFS is not without its limitations (e.g., reliance on a self-report survey), the study represents an important step forward in the movement toward building “global well-being science” [3]. Between the expansive conceptual scope of human flourishing that undergirds the GFS survey, the cultural and geographic coverage of the GFS, and open science approach that has been applied to the study, the GFS could serve as a pivotal resource for enriching existing knowledge about human flourishing in many different parts of the world. More broadly, the well-documented process by which the GFS was developed also provides a valuable template that other scholars could draw on in the development and execution of future global studies [36].

### Commitment to open science practices

Data for Wave 1 of the GFS is available through the Center for Open Science upon submission of a pre-registration and will be openly available without pre-registration beginning February 2025. Subsequent waves of the GFS will similarly be made available. Please see https://www.cos.io/gfs-access-data for more information about data access.

## References

[1] VanderWeele TJ. On the promotion of human flourishing. PNAS. 2017;114:8148–56. 10.1073/pnas.1702996114. 28705870 10.1073/pnas.1702996114 PMC5547610

[2] Lomas T, VanderWeele TJ. The garden and the orchestra: generative metaphors for conceptualizing the complexities of well-being. Int J Environ Res Public Health. 2022;19:14544. 10.3390/ijerph192114544. 36361423 10.3390/ijerph192114544 PMC9657769

[3] Lomas T. Making waves in the great ocean: a historical perspective on the emergence and evolution of wellbeing scholarship. J Posit Psychol. 2022;17:270–5. 10.1080/17439760.2021.2016900.

[4] Henrich J, Heine SJ, Norenzayan A. Most people are not WEIRD. Nature. 2010;466:29. 10.1038/466029a. 20595995 10.1038/466029a

[5] Ghai S. It’s time to reimagine sample diversity and retire the WEIRD dichotomy. Nat Hum Behav. 2021;5:971–2. 10.1038/s41562-021-01175-9. 34285391 10.1038/s41562-021-01175-9

[6] Wong PTP, Cowden RG. Accelerating the science and practice of psychology beyond WEIRD biases: enriching the landscape through Asian psychology. Front Psychol. 2022;13:1054519. 10.3389/fpsyg.2022.1054519. 36619071 10.3389/fpsyg.2022.1054519 PMC9815563

[7] Höltge J, Cowden RG, Lee MT, Bechara AO, Joynt S, Kamble S, Khalanskyi VV, Shtanko L, Kurniati NMT, Tymchenko S, Voytenko VL, McNeely E, VanderWeele TJ. A systems perspective on human flourishing: exploring cross-country similarities and differences of a multisystemic flourishing network. J Posit Psychol. 2022;18:695–710. 10.1080/17439760.2022.2093784.

[8] Johnson BR, Ritter Z, Fogleman A, Markham L, Stankov T, Srinivasan R, Honohan J, Ripley A, Philips T, Wang H, VanderWeele TJ. The Global Flourishing Study; 2024. 10.17605/OSF.IO/3JTZ8

[9] Ritter Z, Srinivasan R, Han Y, Chattopadhyay M, Honohan J, Johnson BR, VanderWeele TJ. Global Flourishing Study methodology. Gallup Inc.; 2024. [Retrieved on [2024-05-10] from https://osf.io/k2s7u].

[10] Crabtree S, English C, Johnson BR, Ritter Z, VanderWeele TJ. Global Flourishing Study: Questionnaire Development report. Gallup Inc; 2021. [Retrieved on [2024-05-10] from https://osf.io/y3t6m]

[11] Lomas T, Cowden RG, Case B, Fogelman A, Johnson BR, VanderWeele TJ. The development of the Global Flourishing Study survey: charting the evolution of a new 109-Item inventory of human flourishing. in prep/under review (to be part of the Nature-Springer-BMC Special Collection). 10.1186/s44263-025-00139-9 PMC12042518 40307930

[12] Wickham H. ggplot2: elegant graphics for data analysis. New York: Springer; 2016.

[13] R Core Team. R: A language and environment for statistical computing. R Foundation for Statistical Computing; 2024. https://www.R-project.org.

[14] Mohler P, Dorer B, de Jong J, Hu M. Cross-cultural survey guidelines. 2016. [Retrieved on [2024-05-06] from https://ccsg.isr.umich.edu/chapters/translation/overview/]

[15] Ritter Z, Markham L, Tyner A, Stankov T, Wang H, Call M, Olson EL, Staller A, Johnson BR, Fogleman A, Ripley A, Phillips T, Srinivasan R, Honohan J, VanderWeele TJ. GFS Translation Document. Center For Open Science; 2024. [Retrieved on [2024-05-06] from https://osf.io/d4qw8].

[16] McPhee C, Barlas F, Brigham N, Darling J, Dutwin D, Jackson C, Jackson M, Kirzinger A, Little R, Lorenz E, Marlar J, Mercer A, Scanlon PJ, Weiss S, Wronski L. Data quality metrics for online samples, Considerations for study design and analysis. American Association for Public Opinion Research; 2022. [Retrieve on [2024-05-07] from https://aapor.org/wp-content/uploads/2023/02/Task-Force-Report-FINAL.pdf].

[17] Edwards JR. Response invalidity in empirical research: causes, detection, and remedies. J Oper Manag. 2019;65:62–76. 10.1016/j.jom.2018.12.002.

[18] Greszki R, Meyer M, Schoen H. The impact of speeding on data quality in nonprobability and freshly recruited probability-based online panels. In: Callegaro M, Baker R, Bethlehem J, Goritz AS, Krosnick JA, Lavrakas PJ, editors. Online panel research: a data quality perspective. Hoboken: Wiley; 2014.

[19] Elliott MR, Valliant R. Inference for nonprobability samples. Stat Sci. 2017;32:249–64.

[20] Frankel MR, Battaglia MP, Link M, Mokdad AH. Integrating cell phone numbers into Random Digit-Dialed (RDD) landline surveys. In: ASA Proceedings of the Social Statistics Section. 2007; 3793–800. http://www.asasrms.org/Proceedings/y2007/Files/JSM2007-000200.pdf

[21] Waksberg J. Sampling methods for random digit dialing. J Am Stat Assoc. 1978;73:40–6. 10.1080/01621459.1978.10479995.

[22] Valliant R, Dever JA, Kreuter F. Practical tools for designing and weighting survey samples. Cham: Springer; 2018.

[23] Kish L. Cumulating/combining population surveys. Surv Methodol. 1999;25:129–38.

[24] Kish L. Methods for design effects. J Off Stat. 1995;11:55–77.

[25] Demographic and Health Surveys Program. Nigeria—National Demographic and Health Data; 2023. [Retrieved from https://data.humdata.org/dataset/dhs-data-for-nigeria].

[26] Valliant R. Comparing alternatives for estimation from nonprobability samples. J Surv Stat Methodol. 2020;8:231–63.

[27] Wiśniowski A, Sakshaug J, Ruiz DAP, Blom A. Integrating probability and nonprobability samples for survey inference. J Surv Stat Methodol. 2020;8:120–47.

[28] Dever JA, Rafferty A, Valliant R. Internet surveys: Can statistical adjustments eliminate coverage bias? Surv Res Methods. 2008;2:47–62.

[29] Sakshaug JW, Wiśniowski A, Perez Ruiz DA, Blom AG. Supplementing small probability samples with nonprobability samples: a Bayesian approach. J Off Stat. 2019;35:629–53.

[30] American Association for Public Opinion Research (AAPOR). Standard definitions: final dispositions of case codes and outcome rates for surveys. 10th edition; 2023.

[31] Peytchev A. Consequences of survey nonresponse. Ann Am Acad Pol Soc Sci. 2013;645:88–111. 10.1177/0002716212461748.

[32] Peytcheva E, Groves RM. Using variation in response rates of demographic subgroups as evidence of nonresponse bias in survey estimates. J Off Stat. 2009;25:193–201.

[33] Dutwin D, Buskirk T. Telephone sample surveys: Dearly beloved or nearly departed? Trends in survey errors in the era of declining response rates. J Surv Stat Methodol. 2021;9:353–80. 10.1093/jssam/smz044.

[34] Groves RM. Nonresponse rates and nonresponse bias in household surveys. Public Opin Q. 2006;70:646–75. 10.1093/poq/nfl033.

[35] Groves RM, Peytcheva E. The impact of nonresponse rates on nonresponse bias: a meta-analysis. Public Opin Q. 2008;72:167–89. 10.1093/poq/nfn011.

[36] Cowden RG, Skinstad D, Lomas T, Johnson BR, VanderWeele TJ. Measuring wellbeing in the Global Flourishing Study: insights from a cross-national analysis of cognitive interviews from 22 countries. Qual Quant. 2024. 10.1007/s11135-024-01947-1.

